# Delayed emergence after general anesthesia: working through the differential diagnosis

**DOI:** 10.1097/MS9.0000000000003360

**Published:** 2025-05-12

**Authors:** Vladislav Zhitny, Elie Geara, Jeffrey Bernstein, Brett Dixon, Ryan Jannoud, Michael Wajda, Yunjia Zhang, Jonathan Alabre, Mohamad Ayoub

**Affiliations:** a Department of Anesthesiology, Perioperative Care, and Pain Medicine, New York University, New York City, New York, USA; b Kirk Kerkorian School of Medicine, University of Nevada, Las Vegas, Nevada, USA

**Keywords:** delayed emergence, general anesthesia, postoperative complications

## Abstract

**Introduction and importance:**

Delayed emergence (DE) following general anesthesia is a critical postoperative complication characterized by the patient’s inability to regain an appropriate level of consciousness 30–60 minutes after surgery. Rapid identification and management of potential causes, including metabolic disturbances and pharmacologic effects, are essential to prevent adverse outcomes.

**Case presentation:**

We present the case of a 67-year-old female with multiple comorbidities, including chronic obstructive pulmonary disease, hemochromatosis, and hypothyroidism, who underwent emergent exploratory laparoscopy for a pelvic abscess. Despite uneventful hemodynamic stability and appropriate anesthetic management, the patient experienced delayed emergence. Initial interventions included reversal agents for neuromuscular blockade and opioids; however, the patient’s mental status continued to fluctuate. Arterial blood gas analysis revealed hypercapnic respiratory metabolic acidosis, prompting ventilatory support and subsequent reintubation. Neurovascular events were ruled out with unremarkable imaging findings.

**Clinical discussion:**

Delayed emergence can result from a variety of etiologies, including residual anesthetic effects, metabolic imbalances, and neurologic events. This case highlights the importance of prompt identification of hypercapnic respiratory metabolic acidosis as a reversible cause of DE. Management included ventilatory support with bilevel positive airway pressure and subsequent intubation, which resolved the acidosis and restored consciousness.

**Conclusion:**

This case underscores the need for a systematic approach in the differential diagnosis of delayed emergence. Timely recognition and management of hypercapnic respiratory metabolic acidosis through ventilatory support were crucial in preventing further complications.

## Introduction

According to the National Institutes of Health, approximately 60 000 people undergo general anesthesia (GA) for surgery each day in the United States^[1]^. While GA is a critical component of modern surgical care, facilitating unconsciousness, analgesia, and muscle relaxation, it is not without risks. Among the numerous potential complications associated with GA, hypoactive emergence, more commonly known as delayed emergence (DE), is a particularly concerning phenomenon. DE refers to the patient’s inability to regain an appropriate level of consciousness, leaving them unresponsive or deeply sedated beyond the expected recovery period, typically 30–60 minutes after the conclusion of GA^[2,3]^.

**HIGHLIGHTS**

- Identification of delayed emergence in anesthesia management: Describes the clinical presentation of delayed emergence (DE) following general anesthesia, emphasizing the need for early identification and intervention. This helps readers understand the importance of recognizing DE as a critical postoperative complication.
- Hypercapnic respiratory metabolic acidosis as a cause of delayed emergence: Highlights the primary diagnosis in this case: hypercapnic respiratory metabolic acidosis, which was identified through arterial blood gas analysis. This emphasizes the metabolic and respiratory factors that can contribute to prolonged unresponsiveness postoperatively.
- Stepwise approach to the differential diagnosis of delayed emergence: Outlines the structured process used in the case, including assessment of anesthetic agents, opioid effects, neuromuscular blockade, and metabolic abnormalities. This provides a systematic framework for other clinicians managing similar cases.
- Role of ventilatory support in resolving hypercapnia: Describes the use of bilevel positive airway pressure and subsequent reintubation as part of managing respiratory acidosis. This emphasizes the importance of respiratory support in the resolution of hypercapnic metabolic acidosis.

The differential diagnosis of DE is broad and includes multiple physiological and pharmacological factors, including residual anesthetic effects, opioid overdose, metabolic derangements, neurologic events, and respiratory insufficiency^[3]^. Given the potential severity of underlying causes, it is important for anesthesia providers to rapidly identify the etiology and intervene accordingly. A structured approach to evaluating DE includes assessing residual anesthetic and neuromuscular blockade, monitoring for metabolic imbalances, and evaluating the patient’s respiratory status to ensure adequate ventilation and gas exchange.

In some cases, delayed emergence can be attributed to rare or overlooked causes, which further complicates timely diagnosis and management. These less commonly recognized factors, such as hypercapnic respiratory acidosis, can also lead to significant alterations in consciousness. Patients with preexisting pulmonary disease, such as chronic obstructive pulmonary disease (COPD), are at particularly high risk for hypercapnia-related complications, making their perioperative management crucial in preventing DE^[4]^.

By highlighting the diagnostic approach, perioperative considerations, and management strategies, this case underscores the importance of maintaining a high index of suspicion for respiratory-driven causes of DE, particularly in patients with underlying pulmonary disease.

## Case report

Here, we present the case of a 67-year-old female with a past medical history of tobacco use, COPD, anxiety, major depressive disorder, hemochromatosis, hypothyroidism, migraines, hypertension, and spinal stenosis. The patient presented with a Computed tomography (CT) scan demonstrating free air and a pelvic abscess near the sigmoid colon, requiring emergent exploratory laparoscopy.

The patient’s thyroid function tests showed a low TSH level of 0.3 mIU/L (reference: 0.4–4.1 mIU/L) with normal free T3 (2.9 pg/mL) and free T4 (1.2 ng/dL).

To address the patient’s history of COPD, the inspiratory-to-expiratory (I:E) ratio was set to 1:1.94, allowing for a prolonged expiratory phase to reduce air trapping and promote effective CO2 elimination.

The patient remained hemodynamically stable throughout the case and emergence. Intra-operative baseline arterial blood gas values were recorded as (7.29pH/44CO2/195 (paO2)/21.2 HCO3). The patient was induced with 50 mcg of fentanyl, 100 mg of propofol, and 60 mg of rocuronium, with a propofol infusion of 60 mcg/kg/min used in conjunction with sevoflurane. Additionally, 0.8 mg of dilaudid was administered for pain control 1 hour prior to the conclusion of the case.

During emergence, it became apparent that the patient was taking more than 30 minutes to become arousable, with inhaled anesthetics at zero and the propofol infusion turned off. Adequate reversal of rocuronium was achieved with sugammadex, and the twitch monitor confirmed sufficient reversal via a train-of-four ratio. Narcan was then administered for opioid reversal. A new arterial blood gas analysis indicated metabolic acidosis with a respiratory acidosis component (7.11pH/65CO2/396 (O2)/20.6 HCO3−) while glucose, potassium, and sodium values remained within normal ranges. The patient’s mental status continued to wax and wane during emergence, with intermittent periods of responsiveness and ability to follow commands followed by sedation.

The surgical intensive care unit (SICU) attending was notified of a potential admission. The SICU attending later came to the operating room to evaluate the patient and discuss a potential plan. As the patient was arousable and otherwise hemodynamically stable, a joint decision was made to trial extubation onto bilevel positive airway pressure (BIPAP) in the post-anesthesia care unit (PACU) to improve ventilation and lower carbon dioxide levels. However, in the PACU, the patient became less responsive and required reintubation due to a declining mental status. A stroke code was called, and the patient was urgently taken for a non-contrast head CT, which showed unremarkable findings.

The patient was subsequently admitted to the SICU, where the admission arterial blood gas analysis indicated hypercapnic respiratory metabolic acidosis (6.98pH/105CO2/131 O2/25.5 HCO3). Following an increase in ventilatory settings, the patient was subsequently successfully extubated and transitioned to nasal cannula. The patient was discharged without any further complications.

## Methods

The patient provided consent for production of this case report. This case report is devoid of any identifiable protected patient information. The case report is exempt from the NYU Langone Institutional Review Board policy. This work has been reported in line with the SCARE 2023 Criteria^[5]^.

## Discussion

In this case, the patient’s delayed emergence was ultimately attributed to hypercapnic respiratory acidosis, a less commonly reported but critical etiology that can lead to altered mental status and further respiratory compromise if not promptly addressed^[4]^. Hypercapnic respiratory acidosis occurs when carbon dioxide retention leads to a significant decrease in pH, impairing neurological function and delaying recovery from anesthesia. Otherwise known as carbon dioxide narcosis, the proposed mechanism is believed to be due to the interruption of central nervous system neurotransmitters involved in consciousness^[6,7]^.

This patient’s arterial blood gas (ABG) values demonstrated worsening hypercapnia (pCO2 65 mmHg in the OR, increasing to 105 mmHg upon SICU admission), correlating with progressive mental status deterioration despite discontinuation of anesthetic agents and opioid reversal. Despite ventilator I:E ratio settings being changed to accommodate, the presence of underlying COPD likely exacerbated CO2 retention, necessitating ventilatory support^[4]^.

To address the patient’s worsening hypercapnia and facilitate CO2 clearance, ventilator settings were adjusted to optimize expiratory time. The I:E ratio was set to 1:1.94, allowing for a prolonged expiratory phase to reduce air trapping and promote effective CO2 elimination, a critical consideration for patients with COPD. A lower I:E ratio is particularly beneficial in obstructive lung disease, as it mitigates dynamic hyperinflation and prevents further respiratory acidosis by ensuring adequate alveolar emptying^[8]^. These ventilatory adjustments were essential in stabilizing the patient’s respiratory status and ultimately enabling a successful extubation.

What makes this case particularly novel is the recognition of severe hypercapnia as the primary driver of DE in a patient who initially appeared stable enough for extubation^[9]^. While DE is often attributed to persistent anesthetic effects or opioid overdose, this case highlights the importance of considering hypercapnic respiratory failure as a critical, reversible etiology^[10]^. Additionally, the fluctuating mental status seen in this patient and the borderline CO2 values contributing to DE underscores the need for serial ABG monitoring and a high index of suspicion for respiratory compromise, even in the absence of immediate hemodynamic instability^[11]^.

This case adds to the growing awareness that DE is not always a purely neurological phenomenon but can be a manifestation of underlying respiratory failure^[12]^. The need for reintubation in the PACU further underscores the importance of cautious extubation planning in high-risk patients, particularly those with preexisting pulmonary disease^[13]^. By forming a strong and quick differential diagnosis and recognizing hypercapnia as a potential contributor to DE, anesthesia providers can enhance postoperative monitoring strategies, optimize ventilatory management, and improve patient outcomes.

The ethics of shared decision-making in this case revolve around balancing patient autonomy, clinical judgment, and the principle of beneficence. Given the patient’s initial responsiveness and hemodynamic stability, the decision to extubate onto BIPAP was made collaboratively between the anesthesia and SICU teams. This approach aimed to optimize respiratory support while minimizing the risks associated with prolonged intubation, such as ventilator-associated pneumonia and airway trauma. However, the subsequent deterioration and need for reintubation in the PACU highlight the complexities of assessing readiness for extubation, particularly in high-risk patients with underlying respiratory disease. These challenges are compounded by the dynamic nature of postoperative physiology, where patients that seem stable can rapidly decompensate due to factors like residual anesthetic effects, impaired airway reflexes, and evolving hypercapnia. This case underscores the ethical responsibility of providers to carefully weigh these risks, engage in interdisciplinary discussions, and remain vigilant in postoperative monitoring to ensure timely intervention while respecting the patient’s best interests. It also reinforces the importance of shared-decision making frameworks that incorporate clinical reassessment and planning to navigate uncertainties in perioperative care.

## Conclusions

**Figure 1.**
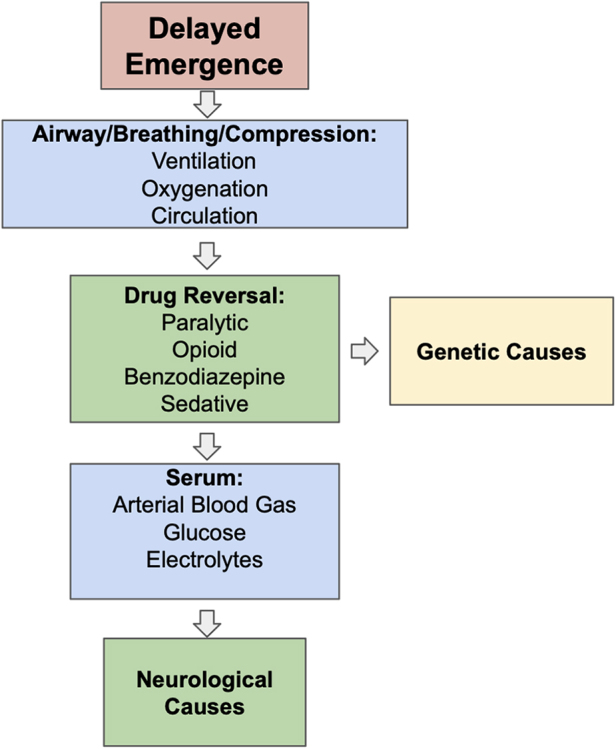
Workflow for differential diagnosis in a delayed emergence patient.

Developing a differential diagnosis in delayed emergence is important for administering appropriate interventions. Possible causes include pharmacokinetic, metabolic, pharmacodynamic, neurologic, or psychiatric factors, as summarized in Table 1. These can involve the use of volatile and intravenous anesthetics, electrolyte imbalances, hypoventilation, opioid overdose, or neurovascular events. Workflow for differential diagnosis in a delayed emergence patient is summarized in Fig. 1. In this case, the patient’s delayed emergence was attributed to hypercapnic respiratory metabolic acidosis.

**Table 1:** Main causes of delayed emergence from general anesthesia

Metabolic/electrolyte abnormalities	Acidosis
	Hyponatremia
	Hypernatremia
	Hypoglycemia
	Hyperglycemia
Neurological abnormalities	Intracranial hemorrhage
	Thrombosis
	Hypoperfusion
	Central anticholinergic syndrome
Psychiatric abnormalities	Conversion disorder
Pharmacokinetic factors	Drug interactions
	Duration of surgery
	Inappropriate dose
	Abnormal transport/distribution
	Abnormal metabolism
	Abnormal elimination
Pharmacodynamic factors	Hypothermia
	Drug interactions
	Genetic variation

### Ventilatory support

Endotracheal intubation and mechanical ventilation should be maintained if end-tidal CO2 is elevated, aiming for a physiological normal level of 40 mmHg. Clearing any volatile anesthetic agents with 100% oxygen should also be part of management.

### Opioid and sedative overdose

A physical exam, including pupillary size and light reflex, can indicate opioid overdose. Consider the opioid’s onset and pharmacokinetics. Naloxone (0.04–0.08 mg) should be given if opioid overdose is suspected. Flumazenil (0.2 mg) may be used if a benzodiazepine overdose is involved, particularly with long-lasting agents.

Consider the half-life of anesthetic agents like propofol, dexmedetomidine, and opioids, used in total intravenous anesthesia. Pre-operative planning should involve discontinuing adjuvant anesthetics if extubation is intended postoperatively.

### Paralysis

Assess paralytic half-life and metabolism, especially in cases of prolonged effects. Rare conditions like pseudocholinesterase deficiency should also be considered, with sedation maintained until spontaneous recovery from neuromuscular blockade occurs.

### Serum electrolytes

Serum electrolyte and glucose imbalances, particularly hypoglycemia, can contribute to altered mental status and should be ruled out in delayed emergence.

### Surgical considerations

The surgical team should confirm the total dose of any medications used intraoperatively. Transparent communication ensures better patient care.

### Neurovascular considerations

Anesthesia can mask intraoperative stroke symptoms. Monitoring for hypotension and arrhythmic events like atrial fibrillation is crucial. A non-contrast head CT scan may be warranted if other causes are ruled out, minimizing radiation exposure and costs. However, reversible causes should be addressed first, guiding the diagnostic and management approach. If ruled out, a psychogenic cause like conversion disorder can be considered.

## Data Availability

Data sharing is not applicable to this article.
